# A Practical Treatise on Nervous Diseases

**Published:** 1904-10

**Authors:** 


### BOOK REVIEWS.

A Practical Treatise on Nervous Diseases. For the Medical Student and General Practitioner. By F. Savary Pearce, M.D., Professor of Nervous and Mental Diseases in the Medico-Chirurgical College, Philadelphia. Octavo, pp. 422. Illustrated. New York and London: D Appleton & Co. 1904. (Price, $3.00.)

The monument which F. Savary Pearce erected to his memory and in the upbuilding of which he lost his own life, will stand long after the marble shaft at his tomb has crumbled and disintegrated. Taken off, the result of a complete nervous breakdown from overwork just as his textbook was being received and applauded by his fellow practitioners, Dr. Pearce, like many others, could not see the beneficent result of his labors. It remains for those coming after to judge of the man by his work and no better illustration of Dr. Pearce's endeavors can be found than this treatise completed a short time before his death.

The author's purpose was to prepare a work which would elucidate the known facts of neurology and curtail or omit altogether all doubtful points in this most intricate science. To learn and remember well grounded facts is difficult enough, but to learn and relearn contradictory and uncertain theories is discouraging and demoralising to the student and general practitioner. On this point the author takes a decided stand and the result is seen on every page of the book.

The opening chapters are devoted to the anatomy and physiology of the nervous system, and the two are happily blended so that the student cannot but have a pretty clear understanding of the structure and functions of the component parts of the nervous system. The neuron theory is excellently described and illustrated.

The next following chapters on pathology, symptomotology and general therapeutics and prevention of nervous diseases are carefully written and must convey to the reader clear understanding of these important branches of neurology. The author goes quite deeply into the consideration of the reflexes—a subject little understood among the general practitioners. The description of therapeutic measures as massage, electricity, diet, and gymnastic exercises is an admirable piece of work and deserves careful consideration by the reader. Although brief, still the more essential points are brought out in a most excellent manner.

The chapters on diseases of the peripheral, cranial and spinal nerves are carefully considered and the treatment especially dwelt upon, a feature of this work and one to be applauded. The etiology of many of these conditions are traced to the very latest literature on the subject. In Landry's disease, for instance, the cause is said to be in all probability an autochthonous poison and some cases are reported having been cured.

The chapters on diseases of the brain and spinal cord are also up to date and presented in a clear, definite manner, so characteristic of the author. Many excellent illustrations are found interspersed through these chapters, the majority of which are from the author's own cases, presenting to the eye a method of teaching that cannot be well explained in the text. The remaining chapters are devoted to the consideration of the haptoneuroses, functional diseases, disorders of sleep and a formulary.

This work can be cheerfully recommended to the student and busy practitioner as one of the best short treatises on the subject.

